# The rs2237892 Polymorphism in *KCNQ1* Influences Gestational Diabetes Mellitus and Glucose Levels: A Case-Control Study and Meta-Analysis

**DOI:** 10.1371/journal.pone.0128901

**Published:** 2015-06-03

**Authors:** Deng Ao, Hai-jun Wang, Li-fang Wang, Jie-yun Song, Hui-xia Yang, Yan Wang

**Affiliations:** 1 Department of Child, Adolescent and Women’s Health, School of Public Health, Peking University, Beijing, China; 2 Key Laboratory of Reproductive Health, Ministry of Health, Beijing, China; 3 Department of Obstetrics and Gynecology, Peking University First Hospital, Beijing, China; University of Catanzaro Magna Graecia, ITALY

## Abstract

**Objective:**

Recent genetic studies have shown that potassium voltage-gated channel, KQT-like subfamily, member1 (*KCNQ1*) gene is related to gestational diabetes mellitus (GDM). However, studies for the rs2237892 polymorphism in *KCNQ1* and GDM remain conflicting in Asians. Furthermore, associations of this polymorphism with glucose levels during oral glucose tolerance test (OGTT) have not been described in Chinese pregnant women. The present study aimed to provide evidence for the associations of rs2237892 in *KCNQ1* with GDM and glucose levels, and to systematically evaluate the effect of rs2237892 on GDM in Asians.

**Methods:**

A case-control study on 562 women with GDM and 453 controls was conducted in Beijing, China. The association of rs2237892 with risk of GDM was analyzed using logistic regression. The associations with quantitative glucose levels were assessed using linear regression models. A meta-analysis including the present case-control study and four previously published reports in Asians was conducted.

**Results:**

The rs2237892 polymorphism in *KCNQ1* was associated with GDM (OR (95%CI) =1.99(1.26-3.15)). Additionally, the polymorphism was associated with levels of 1h and 2h glucose during OGTT. The pre-pregnancy BMI, age and genotypes of *KCNQ1* polymorphism were independent risk factors of GDM. Subsequently, we performed a meta-analysis in Asians. In total, C-allele carriers of rs2237892 polymorphism had a 50% higher risk for GDM (OR (95%CI) =1.50(1.15-1.78)).

**Conclusion:**

The study demonstrated for the first time that the *KCNQ1* rs2237892 polymorphism was associated with GDM and glucose levels in Chinese women. The study provides systematic evidence for the association between this polymorphism and GDM in Asians.

## Introduction

Gestational diabetes mellitus (GDM) is defined as glucose intolerance that first occurs or is first identified during pregnancy [1]. It is a metabolic disease with a continuously increasing prevalence worldwide [2–4]. Also in urban women of China, the prevalence of GDM increased from 2.4 to 12.1% during 1999–2013 [5, 6]. GDM has become an important public health problem in the world. GDM may increase the likelihood of several maternal and perinatal complications [7]. Increased maternal glucose levels also have relation with adverse pregnancy outcomes including delivery by cesarean section, future pre-diabetes or diabetes, neonatal hypoglycemia and adiposity [8–10].

The search for candidate genes for GDM has been active, and more and more genes associated with GDM are being discovered. These genetic factors are also likely to contribute to variation in glucose levels in pregnancy. One of these genes is the maternally expressed gene potassium voltage-gated channel, KQT-like subfamily, member1 (*KCNQ1*) [11]. The *KCNQ1* gene, located on 11p15.5, encodes the pore-forming a-subunit of the voltage-gated K^+^ channel (KvLQT1) [12]. In addition, *KCNQ1* is also expressed in pancreatic islets and the cultured insulin-secreting INS-1 cells, and blockade of the channel with *KCNQ1* inhibitors 293B stimulated insulin secretion [13], suggesting that *KCNQ1* channels may play a role in regulation of insulin secretion.

The rs2237892 polymorphism in *KCNQ1* was first observed to be associated with GDM in a Korean population [14]. The association between rs2237892 and GDM was replicated in a subsequent study in another Korean population [15]. This study also found that rs2237892 was associated with an increased glucose level at 1 and 2 h of 100g oral glucose tolerance test (OGTT) in GDM women. A meta-analysis including only three studies was performed to find the association of rs2237892 and GDM [16]. In this meta-analysis, one study in Chinese population showed that rs2237892 was not associated with the risk of GDM [17]. Recently, a new publication in Chinese population has been published. This recent study also showed that rs2237892 was not associated with GDM [18]. Moreover, the associations of this single nucleotide polymorphism (SNP) with glucose levels during OGTT in pregnancy have not been described in Chinese. We did not find any published study examining the association between rs2237892 and GDM in populations of European. It may due to the less frequency of the minor allele there (4–8% compared to 31–40% in Asians) [14, 15, 17–22]. Meta-analysis is potentially powerful tool combining results across studies to increase the sample size, getting enough power to clarify inconsistent results in genetic association studies [23]. Since previous epidemiological studies with relatively small sample sizes on the association between rs2237892 and GDM showed controversial results, a comprehensive meta-analysis was necessary for investigating the real effect of the *KCNQ1* in risk of GDM.

Therefore, we conducted a case-control study to assess the association of the *KCNQ1* rs2237892 polymorphism with GDM and glucose levels in Chinese women. Subsequently, a meta-analysis including the present case-control study and four previously published reports was conducted to systematically evaluate the contribution of rs2237892 to GDM in Asians.

## Materials and Methods

### Case-control study

#### Study population

The pregnant women aged 20–49 years and at 24–28 gestation weeks were recruited consecutively from the Department of Obstetrics and Gynecology of Peking University First Affiliated Hospital in Beijing from May 2012 to November 2013. The exclusion criteria for subjects were as follows: women who had pre-existing diabetes, or abnormal result in a glucose screening test before the 24th week of gestation, or multiple gestations, or maternal diseases such as hypertension, endocrine disorders, and hepatic diseases; women with incomplete medical information and who had decided to give birth at another hospital; women who were unable or unwilling to get involved in the study. Ultimately, 562 cases and 453 controls were enrolled in this study by providing written informed consent.

All pregnant women were screened for GDM at 24–28 gestation weeks with a 75g, 2h OGTT after overnight fast, according to the criteria established by the Ministry of Health of China in 2011, which is consistent with the guidelines of International Association of Diabetic Pregnancy Study Group [24]. The threshold glucose values were as follows: 5.1mmol/L (92mg/dl) for fasting, 10.0mmol/L (180mg/dl) for 1 hour, and 8.5mmol/L (153mg/dl) for 2 hours. GDM was diagnosed if one or more of glucose values met or exceeded the threshold. The controls were pregnant women having normal glucose tolerant, which was identified by the OGTT test at 24–28 gestation weeks.

The clinical and biochemical data were collected by the trained medical record abstractors from the hospital computer database, which included self-reported pre-pregnancy weight and height measured at the first prenatal visit. The pre-pregnancy BMI was calculated by dividing pre-pregnancy weight in kilograms by height in meters squared. The glucose levels during OGTT were detected by a biochemical automatic analyzer (Hitachi 7600, Tokyo, Japan). The glucose levels were measured to the nearest 0.01mmol/L.

#### Ethics statement

The study adhered to the principles of the Declaration of Helsinki. The study protocol and written informed consent were approved by the institutional review board, Peking University Biomedical Ethics Committee (IRB00001052-12043). We obtained written informed consent from all the study participants.

#### Genotyping

Genomic DNA was extracted from peripheral blood samples by salting-out procedure. SpectroDESIGNER software (Sequenom, San Diego, CA) was used to design the primers, including a pair of amplification primers and an extension primer. Sequenom’s MassARRAY platform (Sequenom, San Diego, CA, USA) was applied for genotyping the rs2237892 polymorphism according to the manufacturer’s instructions [25]. Negative controls and two samples were placed in duplicate on each run, to ensure correct genotyping.

#### Statistical analyses

The quantitative variables with normal distribution was given as mean and standard deviation (SD), and tested by t-test. The chi-square test was used to determine whether the polymorphism was in Hardy-Weinberg equilibrium (HWE). Genotype frequencies in GDM subjects and controls were compared by logistic regression under the dominant model to calculate odds ratios (OR). The ORs were presented with 95% confidence intervals (CIs). The quantitative traits were analyzed by linear regression, and the regression coefficients (b) were presented. A two-sided *P* value of less than 0.05 was considered to be statistically significant. The statistical analyses were performed with SPSS version 18.0 (SPSS Inc, Chicago, IL, USA).

### Systematic review and meta-analysis

We searched the databases of PubMed, Wan fang and Chinese National Knowledge Infrastructure (CNKI) for papers published in English or Chinese before November 2014. Wan Fang database comprises over 30 million journal articles from 7,774 full-text journal titles published in China, covering over 90% of the core journals. Wan Fang database in Medicine and Health Sciences can be a valuable tool for Chinese medical research. The key words of our search included *KCNQ1*, rs2237892 and GDM. Reference lists of retrieved articles were also searched to check for additional reports of relevant studies.

Eligible studies met all the following criteria: (1) original papers; (2) case—control studies; (3) genotype frequencies in cases and controls or odds ratio with its 95% CI were provided. The exclusion criteria were: (1) overlapping data; (2) case-only studies; (3) family-based studies; (4) reviews.

The following data was extracted from each study independently by two reviewers according to a fixed protocol: name of first author, year of publication, ethnic background of study subjects, criteria followed for diagnosis of GDM, source of control, numbers of cases and controls, method of genotyping, HWE status, genotype frequencies in the GDM subjects and controls. In addition, we tried to contact with the corresponding author to get the detailed data if the genotype distribution was unavailable in the article. The quality scoring scale modified from previous meta-analysis of genetic studies was used to evaluate the quality of included studied by two reviewers [26, 27]. The highest quality score was 12 points (S1 Table). The study obtained 7 points or higher score was categorized as “high quality” [26]. All disagreements were discussed between two reviewers.

The meta-analysis was performed with Review Manager version 5.0 (The Cochrane Collaboration, Nordic Cochrane Centre, Copenhagen, Denmark). The data extracted from all publications were tested under the dominant model. The pooled OR with a corresponding 95% CI was calculated to evaluate the association between the rs2237892 polymorphism and GDM. The Z test was performed to determine the significance of the pooled OR. The HWE of the control group was assessed by chi-square test. The heterogeneity was assessed by Q test and *I* ^*2*^ value (*I* ^*2*^ value of 25, 50 or 75% was considered to be low, moderate, or high heterogeneity, respectively [28]).The pooled OR and 95% CI were calculated by fixed effect model if *P* > 0.05 in the Q tests; otherwise they were calculated by random effect model. Funnel plots and Egger’s regression test were used to explore the publication bias [27]. Sensitivity analysis was performed to evaluate the influence of each individual study on the overall results [29].

## Results

### Case-control study

#### General characteristics

The general characteristics of the GDM patients and controls are shown in Table 1. The average age, pre-pregnancy BMI and glucose levels during OGTT were significantly higher in GDM subjects, compared with the controls (*P* <0.001).

**Table 1:** General characteristics of participants.

Category	GDM subjects	Controls	*P* value
N	562	453	—
Age (year)	30.18±2.64	29.50±2.68	<0.001
Pre-pregnancy BMI (kg/m^2^)	22.39±3.26	21.25±2.97	<0.001
OGTT-Fasting plasma glucose (mmol/L)	5.16±0.52	4.55±0.27	<0.001
OGTT-1h glucose (mmol/L)	9.52±1.66	7.37±1.23	<0.001
OGTT-2h glucose (mmol/L)	8.13±1.55	6.43±0.95	<0.001

Abbreviations: N, number; BMI, Body mass index; OGTT, Oral glucose tolerance test.

#### Association of rs2237892 with GDM

In our population, C-allele frequency for rs2237892 was 71.1%, which is higher than the frequency found in Asians (60–69%) [14, 15, 17, 18]. The rs2237892 polymorphism was in HWE in the control group (*P* = 0.883). Genotype frequencies in the GDM subjects and controls are represented in Table 2. Odds ratio was calculated by logistic regression and showed that the C-allele carriers (TC or CC) was significantly associated with GDM (OR (95%CI) = 1.99(1.26–3.15), *P* = 0.003) (Table 2).

**Table 2:** Genotype frequencies of rs2237892 and its odds ratio for GDM.

Group	Genotype n (%)		Unadjusted		Adjusted	
	TT	TC+CC	OR(95% CI) ^*a*^	*P* value ^*a*^	OR(95% CI) ^*b*^	*P* value ^*b*^
Controls	50(11.0)	403(89.0)	1.0	—	1.0	—
GDM subjects	33(5.9)	529(94.1)	1.99(1.26–3.15)	0.003	2.19(1.36–3.54)	0.001

^*a*^ Unadjusted odds ratio with 95% confidence interval (CI) and *P*-value was estimated with logistic regression analysis under dominant model.

^*b*^ Adjusted odds ratio with 95% confidence interval (CI) and *P*-value was estimated with logistic regression analysis adjusted for age and pre-pregnancy BMI under dominant model.

#### Association of rs2237892 with glucose levels

We examined associations between the rs2237892 polymorphism and quantitative glucose levels measured during OGTT, including fasting glucose level, 1h and 2h glucose levels (Table 3). The rs2237892 polymorphism was significantly associated with glucose level at 1h and 2h of OGTT (*P* = 0.004 and 0.027). The mean fasting glucose level was 4.89±0.52mmol/L in the combined TC and CC genotypes and 4.83±0.51mmol/L in the TT homozygotes. The 1h glucose level was significantly higher in C-allele carriers than in the TT homozygotes (8.62±1.85 vs. 8.01±1.72mmol/L). And the C-allele carriers also had higher 2h glucose level (7.41±1.59mmol/L) than the TT homozygotes (7.01±1.31mmol/L).

**Table 3:** Linear regression analyses of the rs2237892 in *KCNQ1* with glucose levels during OGTT.

Category	Genotype (TT)	Genotype (TC+CC)	Regression coefficient (b)	*P* value
OGTT-Fasting plasma glucose (mmol/L)	4.83±0.51	4.89±0.52	0.068	0.257
OGTT-1h glucose (mmol/L)	8.01±1.72	8.62±1.85	0.608	0.004
OGTT-2h glucose (mmol/L)	7.01±1.31	7.41±1.59	0.399	0.027

*P* value was calculated with linear regression analysis under dominant model.

Abbreviations: OGTT, Oral glucose tolerance test.

#### Association of rs2237892, age, and pre-pregnancy BMI with GDM

Since GDM is a multifactor disease, we assessed the correlation of rs2237892 with maternal factors involved in the appearance of GDM, such as age, pre-pregnancy BMI. In the logistic model, high values of pre-pregnancy BMI (OR (95%CI) = 1.11(1.07–1.16); *P*<0.001) and age (OR (95%CI) = 1.09 (1.03–1.14); *P* = 0.001) were independently associated to GDM. Remarkably, the logistic model with age, pre-pregnancy BMI, and genotypes of *KCNQ1* polymorphism as independent variables showed a significant association of C-allele carriers with GDM (OR (95%CI) = 2.19 (1.36–3.54); *P* = 0.001) (Table 2). And we did not identify interaction between rs2237892 and age or pre-pregnancy BMI.

### Systematic review and meta-analysis

**Fig 1:**
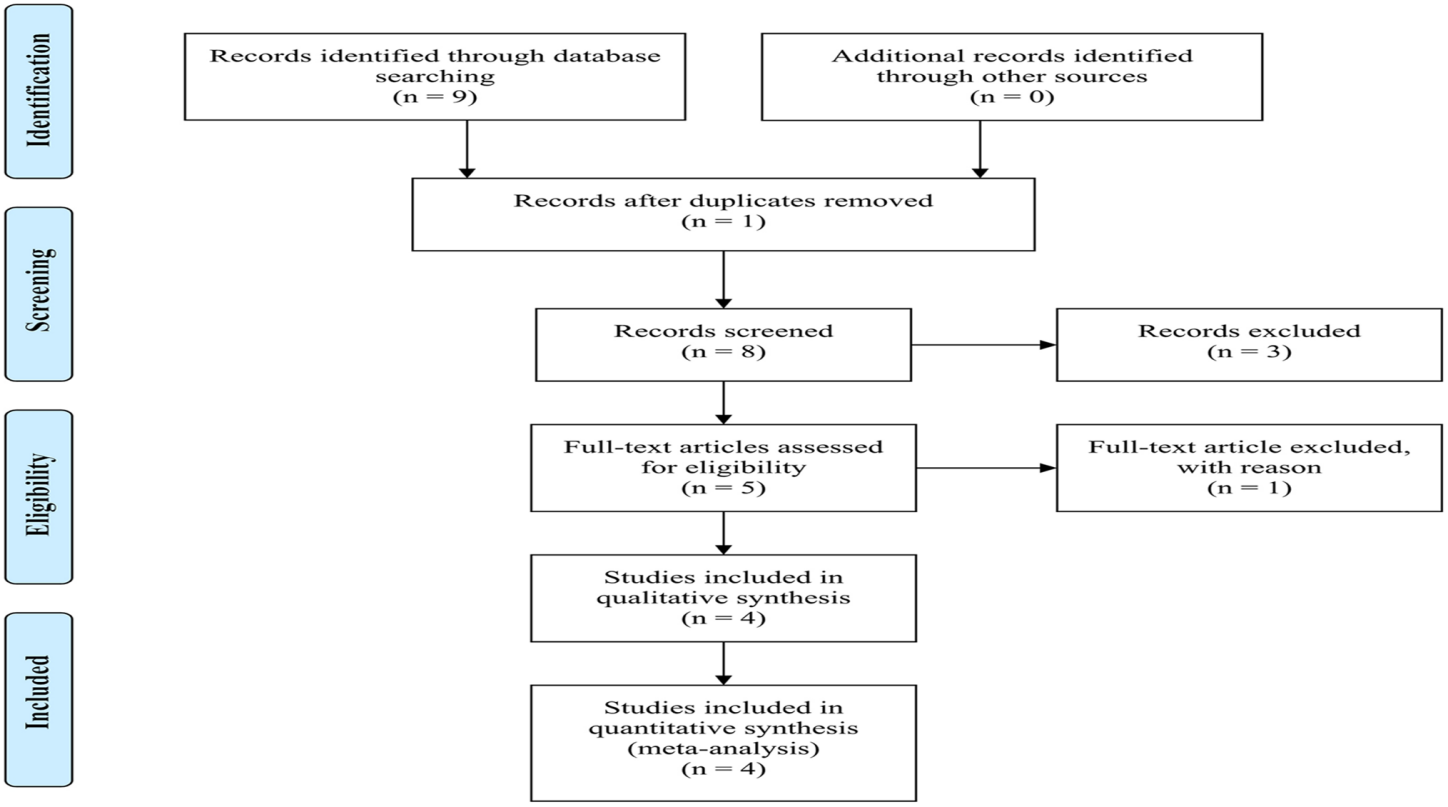
Flow diagram of study selection.

A total of nine articles were identified for *KCNQ1* and GDM by searching three databases. Relevant titles and summaries were identified and five were chosen from nine articles after removing duplicate, reviews and meta-analysis. Of the five full-text articles, one was further excluded for different SNP (Fig 1). Four case-control studies met the inclusion criteria [14, 15, 17, 18]. The detailed characteristics of these studies were presented in Table 4. With respect to the assessment of study quality, all the included studies were high quality (S2 Table).

**Table 4:** Main characteristics of included studies in meta-analysis.

Study	Ethnicity	Study Design	Diagnostic Criteria	Control	Case/Control (n)	Genotyping Method
Zhou 2009	Chinese	Case-Control	ADA (2000)	NGT and 50g GCT negative pregnant women	520/916	PCR-RFLP
Kwak 2010	Korean	Case-Control	Third International Workshop Conference	Non-diabetic subjects	853/624	TaqMan assay
Shin 2010	Korean	Case-Control	Third International Workshop Conference	NGT pregnant women	930/631	TaqMan assay
Zhai 2014	Chinese	Case-Control	ADA (2000)	NGT and 50g GCT negative pregnant women	185/100	PCR-RFLP

Abbreviations: ADA, American Diabetes Association; NGT, Normal glucose tolerance; GCT, Glucose challenge test; PCR-RFLP, Polymerase chain reaction-restriction fragment length polymorphism.

**Fig 2:**
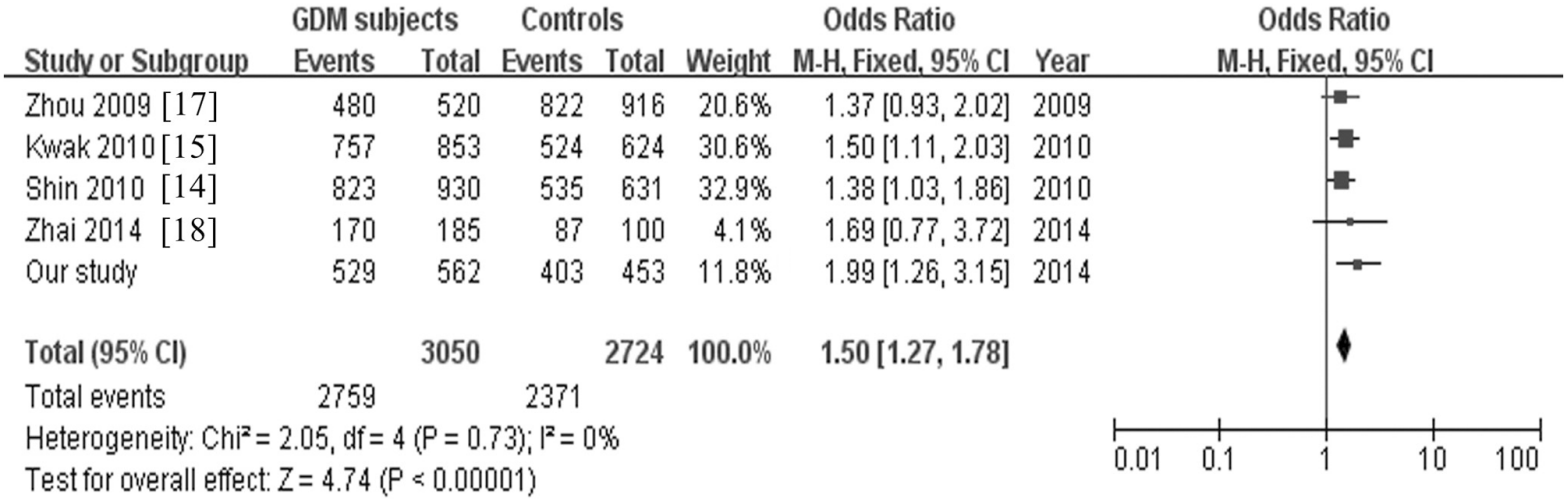
Meta-analysis for the studies of the rs2237892 in *KCNQ1* with GDM.

The four studies and our study involved in the meta-analysis contain a total of 3,050 women with GDM and 2,724 controls. The frequency of C-allele was 66.1%. Using a fixed effect model, the pooled OR of the C-allele carriers for GDM was 1.50 (95% CI: 1.27–1.78, P<0.001) (Fig 2), indicating that the C-allele carriers had a 50% increased risk for GDM. It suggested the positive association between the rs2237892 polymorphism and GDM.

**Fig 3:**
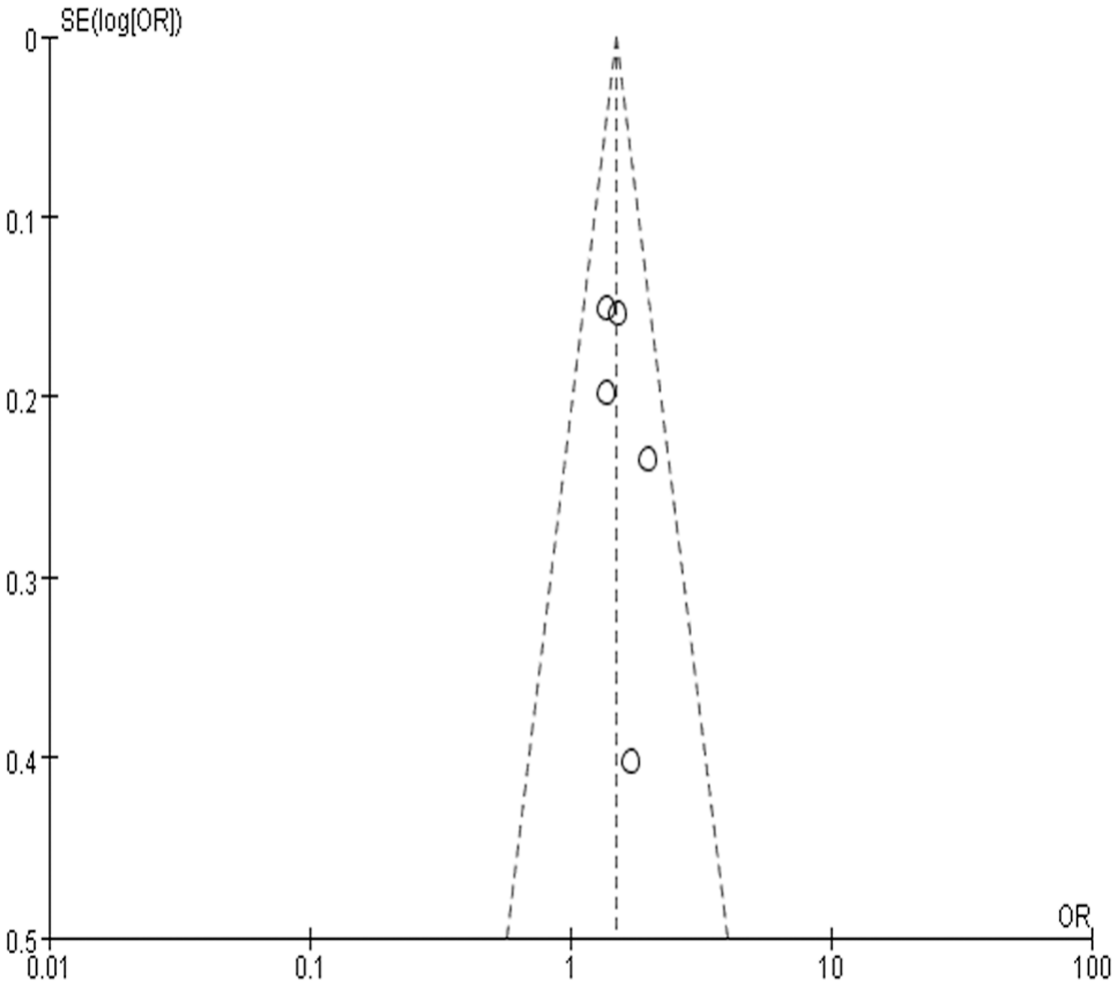
Funnel plot of the five studies. Abbreviations: OR, odds ratio; Log [OR], natural logarithm of OR; SE, standard error; SE (Log [OR]), standard error of Log [OR].

There was no evidence for heterogeneity among these studies (Q = 2.05, *P* = 0.730). The funnel plot for the association between rs2237892 and GDM showed a symmetrical distribution, with *P*-value of Egger’s test 0.349, suggesting no publication bias (Fig 3).

When we performed sensitivity analysis by sequential removal of individual studies, there was no one study that could significantly affect the pooled OR.

## Discussion

In this study, our results showed that *KCNQ1* rs2237892 was significantly associated with the risk of GDM and glucose levels at 1h and 2h measured during OGTT amongst Chinese women. The study reported for the first time in Chinese population the *KCNQ1* rs2237892 polymorphism plays an important role in risk of GDM and increased glucose levels.

*KCNQ1* gene encodes a voltage-gated potassium channel. It plays an important role in cardiac action potential [20]. *KCNQ1* is also expressed in the pancreas, including the pancreatic islets. Previous studies have shown that *KCNQ1* reduced glucose-stimulated insulin secretion [21, 30, 31]. The contribution of the *KCNQ1*-encoded protein to the molecular pathogenesis of GDM remains to be elucidated. In this study, *KCNQ1* rs2237892 SNP was not only associated with a risk for GDM, but also with increased 1h and 2h glucose levels which was consistent with the known association between this SNP and glucose-stimulated insulin secretion. The Hyperglycemia and Adverse Pregnancy Outcome Study showed a continuous positive relationship between glucose levels during OGTT and adverse pregnancy outcomes [9]. The rs2237892 in *KCNQ1* that is associated with steady-state glucose regulation probably has potentially harmful outcomes in pregnancy.

Our results showed age of GDM subjects was significantly higher than controls, which is supported by the previous study showing age was associated with an elevated GDM [32]. Some studies have established a relationship between increasing pre-pregnancy weight and GDM [33, 34]. In our study, the pre-pregnancy BMI of GDM group was significantly higher than that of controls. And pre-pregnancy BMI had a significant OR for GDM. Recently, Yu et al found an interaction of rs2237892 with BMI on type 2 diabetes mellitus in 12,273 Chinese participants (*P* = 0.012) [35]. However, logistic analysis in our study reported that pre-pregnancy BMI and GDM were independently associated to GDM, and there was no interaction between them. All the four studies on rs2237892 and GDM did not explore the interaction between *KCNQ1* genotype and pre-pregnancy BMI. Further studies including more subjects to investigate the role of this SNP will be needed.

Two published reports in Chinese population showed that *KCNQ1* polymorphism (rs2237892) was not associated with GDM [16, 17]. However, our case-control study revealed a significant association, which was similar with two published reports in Korean population [14, 15]. The inconsistent findings might be due to the relatively small sample size in these studies including our study. A meta-analysis can use a statistical approach to combine both statistically significant and non-significant results and provide more convincing estimates of effect in genetic association studies [36]. With lack of meta-analysis, we performed a meta-analysis in 5,774 women of five studies (including the present study), including 3,050 GDM cases and 2,724 controls. We found that women with C-allele have a 50% increased risk to develop GDM. There was no significant heterogeneity among all studies in our meta-analysis. Moreover, sensitivity analysis showed that no single study qualitatively affected the pooled OR, suggesting the highly stability of the results. The meta-analysis confirmed the significant association between rs2237892 polymorphism and GDM. It indicated that our finding in case-control study was unlikely by chance.

There was a single GDM-GWAS performed in Korean population [37], in which *KCNQ1* variants (rs163184 and rs231362) were not associated with GDM. Considering the two variants were in weak linkage disequilibrium with rs2237892 of the present study (*r* ^*2*^ = 0.435 between rs163184 and rs2237892 and *r* ^*2*^ = 0.000 between rs231362 and rs2237892 in Han Chinese in Beijing, China), the different regions of *KCNQ1* gene might be functionally different. Further fine mapping investigations are needed to clarify which part of *KCNQ1* gene is responsible for the potential GDM association.

There were several limitations of the meta-analysis. First, we only assessed rs2237892 polymorphism in *KCNQ1* gene, therefore, we cannot rule out the possibility that other polymorphisms or haplotypes in this gene might be also implicated in the development of GDM. Second, lack of individual-level data on lifestyle factors, such as diet and physical activities, prevents us from analyzing any interaction between rs2237892 and other factors on GDM. A study showed that diet with low fiber and high glycemic load was associated with GDM [38]. Physical activity was reported to reduce the risk of GDM [39]. Further study should consider the potential gene-environment interactions. Third, GDM patients were selected at a third-tier hospital of Beijing and thus may have been unrepresentative of the general patients in China. Notably, the control subjects were recruited at the same hospital. In addition, the rs2237892 polymorphism was in HWE in the control group, suggesting the randomness of subject selection.

In conclusion, the rs2237892 polymorphism in *KCNQ1* was identified to be associated with risk of GDM and glucose levels by the case-control study and meta-analysis. The rs2237892 polymorphism could provide clues to forecast susceptibility to GDM in Chinese population. Further studies should be conducted to clarify the functional significance of the *KCNQ1* rs2237892 polymorphism.

## Supporting Information

S1 Checklist PRISMA checklist. (DOC)

S2 Checklist Genetic Meta Analysis checklist. (DOC)

S1 Table Scale for quality assessment. (DOC)

S2 Table Quality score assessment results. (DOC)
