# Supplementary material for: The rs2237892 Polymorphism in KCNQ1 Influences Gestational Diabetes Mellitus and Glucose Levels: A Case-Control Study and Meta-Analysis
Source: PLoS One. 2015 Jun 3;10(6):e0128901. doi: 10.1371/journal.pone.0128901 (PMC4454508; doi:10.1371/journal.pone.0128901)
Supplement: S1 Table — (DOC) [file pone.0128901.s003.doc]

**S1** Table: Scale for quality assessment

| **Criteria** |  | **Score** |
| --- | --- | --- |
| Representativeness of cases | Consecutive/randomly selected from case population with clearly defined sampling frame | 2 |
|  | Consecutive/randomly selected from case population without clearly defined sampling frame | 1 |
|  | Not described | 0 |
| Source of controls | Population- or neighbor-based | 2 |
|  | Hospital-based | 1 |
|  | Not described | 0 |
| Hardy-Weinberg equilibrium in controls | Hardy-Weinberg equilibrium | 2 |
|  | Hardy-Weinberg disequilibrium | 1 |
| Genotyping examination | Genotyping done under “blinded” condition | 1 |
|  | Unblinded or not mentioned | 0 |
| Association assessment | Assess association between genotypes and GDM with appropriate statistics and adjustment for confounders | 2 |
|  | Assess association between genotypes and GDM with appropriate statistics without adjustment for confounders | 1 |
|  | Inappropriate statistics used | 0 |
| Total sample size | ≥1000 | 3 |
|  | ≥500 but <1000 | 2 |
|  | ≥200 but <500 | 1 |
|  | <200 | 0 |
| Total |  | 12 |
