# Supplementary material for: The rs2237892 Polymorphism in KCNQ1 Influences Gestational Diabetes Mellitus and Glucose Levels: A Case-Control Study and Meta-Analysis
Source: PLoS One. 2015 Jun 3;10(6):e0128901. doi: 10.1371/journal.pone.0128901 (PMC4454508; doi:10.1371/journal.pone.0128901)
Supplement: S2 Table — (DOC) [file pone.0128901.s004.doc]

**S2** Table. Quality score assessment results

| **Study** | **Representativeness of case** | **Source of controls** | **Hardy-Weinberg equilibrium in controls** | **Genotyping examination** | **Association assessment** | **Total sample size** | **Total Score** |
| --- | --- | --- | --- | --- | --- | --- | --- |
| Zhou 2009 | 1 | 1 | 2 | 0 | 2 | 3 | 9 |
| Kwak 2010 | 1 | 1 | 2 | 0 | 2 | 3 | 9 |
| Shin 2010 | 1 | 1 | 2 | 0 | 2 | 3 | 9 |
| Zhai 2014 | 1 | 1 | 2 | 0 | 2 | 1 | 7 |
